# Reflections on the production and use of interviews as a learning tool in educational podcasts for medical undergraduates

**DOI:** 10.1192/j.eurpsy.2022.2191

**Published:** 2022-09-01

**Authors:** S. James, T. Awolesi, S. De Souza

**Affiliations:** 1 University of Bristol, Medical School, Bristol, United Kingdom; 2 Somerset NHS Foundation Trust, Pyrland Ward, Wellsprings Hospital, Taunton, United Kingdom

**Keywords:** podcast, medical students, undergraduate, interviewing

## Abstract

**Introduction:**

The therapeutic interview is one of the cornerstones of psychiatric practice. When practitioners are skilled in the art of interviewing, patients are allowed to share their narratives and explore their emotions, while the clinician can diagnose and treat more effectively. Discussions with colleagues can be used to share knowledge and experience. As part of a student project undertaken in Summer 2021, two students were tasked with producing a series of educational podcasts to be used for psychiatric training. Both students chose to complete several interviews with professionals in psychiatry. In this study, they will reflect on the efficacy of interviews as a learning tool, their experiences of podcast interviewing, and the transferable skills that they learned.

**Objectives:**

To reflect on the use of interviews within podcasts and how this can be transferred to practice.

**Methods:**

Based on the literature review, medical students conducted interviews with specialist clinicians in order to gain information around patient treatment. They then reflected on this experience.

**Results:**

The practice of interviewing allowed for the development of a range of skills. It improved communication with senior professionals, provided a deeper knowledge of different psychiatric fields, and developed the written skills needed for the creation of engaging questions.

**Conclusions:**

Interviews are a useful tool in educational podcasts and producing these helped medical students gain specialist insight and learning into different medical fields not thoroughly covered in the undergraduate curriculum. Producing the podcasts helped to develop the key skill of interviewing and communicating with more senior colleagues.

**Disclosure:**

No significant relationships.

